# Left ventricular hypertrophy and myocardial work in amateur marathon runners

**DOI:** 10.3389/fcvm.2025.1707542

**Published:** 2025-12-04

**Authors:** Juncheng Wang, Haiyan Tian, Qinghong Zeng, Sheng Cao, Yanggan Wang

**Affiliations:** 1 Department of Geriatrics and General Medicine, Zhongnan Hospital of Wuhan University, Wuhan, China; 2 Department of Internal Medicine, Nanchang Hongdu Hospital of Traditional Chinese Medicine, Nanchang, China; 3 Department of Ultrasound, Renmin Hospital of Wuhan University, Wuhan, China

**Keywords:** amateur marathon runner, left ventricular hypertrophy, myocardial work, pressure-strain loop, sports cardiology

## Abstract

**Aims:**

To investigate the left ventricular hypertrophy (LVH) and myocardial work, and their association in amateur marathon runners.

**Methods:**

Amateur marathon runners were categorized into LVH and non-LVH groups according to international guidelines and further classified by gender. Healthy individuals without established exercise habits served as the control group. The global work index (GWI), global constructive work (GCW), global work efficiency (GWE), and global wasted work (GWW) were calculated using the pressure-strain loop methodology. The Firth logistic regression model was used to analyze the factors influencing LVH. Spearman correlation analysis was utilized to examine the relationship between running characteristics and myocardial work parameters.

**Results:**

Compared with the control group, both the non-LVH and LVH groups exhibited significant enlargement of the left atrium and left ventricle (LV), increased wall thickness, and elevated LV mass, with the LVH group showing more pronounced changes. The non-LVH group demonstrated the highest GWI and GCW, while the LVH group exhibited the lowest GWE and GWW. Regression analysis indicated that the weekly running distance is associated with LVH (OR = 1.97, CI: 1.37–3.48) in male runners. In the overall runner cohort, the weekly running distance was negatively correlated with GWE (*r* = −0.37, *P* = 0.002) and positively correlated with GWW (*r* = 0.34, *P* = 0.004).

**Conclusion:**

Excessive remodeling leading to LVH may be associated with decreased myocardial work efficiency in amateur marathon runners. The weekly running distance may be a factor influencing LVH and myocardial work.

## 1 Introduction

With the increasing prevalence of amateur marathon running, it has become imperative to understand its effects on myocardial performance (1). While it is widely accepted that exercise training enhances cardiac function (2, 3), some studies indicate that marathon running may induce cardiac fatigue (4). Currently, there is no definitive consensus regarding the impact of marathon running on cardiac function (5, 6), which may be associated with the degree of left ventricular (LV) remodeling. Mitchell et al. (7) classify distance running as a high-dynamic, low-static activity, which may increase the volume load, leading to greater LV mass and chamber size (eccentric hypertrophy). The exercise volume of amateur marathon runners falls between that of the general population and professional athletes. Their LV hypertrophy (LVH) and cardiac function warrant further investigation.

Global longitudinal strain (GLS) is widely employed to evaluate LV function in runners (8); however, it is influenced by afterload (9). The pressure-strain loop is an innovative method for quantifying myocardial work and addresses the load dependency of GLS (10). The pressure-strain loop offers a superior approach for assessing myocardial function, providing deeper insights into myocardial energy metabolism and oxygen consumption (11).

Our study aims to investigate LVH and myocardial work, and their association, in amateur runners. We hypothesize that not all amateur runners will exhibit LVH, and that LVH may be associated with impaired LV contraction. This research contributes to a deeper public understanding of exercise cardiology and promotes cardiovascular health among amateur marathon runners.

## 2 Methods

### 2.1 Study population

Between January 2024 and September 2025, 68 amateur marathon runners were recruited from various local marathon clubs, including 26 females. The inclusion criteria for amateur marathon runners were as follows: 1) Participation in marathon running for at least one year and completion of at least one marathon; 2) A running training frequency exceeding three times per week, with each running session covering a distance of at least 10 kilometers; 3) No participation in any marathon races within the two weeks preceding the examination; 4. No history of medication use that could potentially affect cardiac function. Based on the LVH standards recommended by the guidelines (12), the runners were categorized into a non-LVH group (males: LVMI ≤ 115 g/m^2^, females: LVMI ≤ 95 g/m^2^) and an LVH group (males: LVMI > 115 g/m^2^, females: LVMI > 95 g/m^2^). Additionally, 40 healthy individuals were recruited from the hospital's health management department and the surrounding community to serve as a control group. The inclusion criteria for healthy individuals were as follows: 1. Engaged in long-term sedentary work; 2. No prior exercise training habits. Basic information about these healthy individuals was obtained through face-to-face interviews. The exclusion criteria for all participants were: 1) Congenital heart disease, valvular heart disease, pericardial diseases, and other cardiac conditions; 2) Hypertension, diabetes, kidney diseases, or other systemic diseases; 3) Pregnant women and children; 4) Poor quality of ultrasound images. This study was conducted in accordance with the Declaration of Helsinki and received approval from the Ethics Committee of Zhongnan Hospital of Wuhan University (No. 2023177). All participants provided informed consent.

### 2.2 Transthoracic echocardiography measurement

The experienced echocardiographers acquired echocardiographic images utilizing the GE Vivid E95 ultrasound machine. Non-invasive cuff blood pressure measurements were performed concurrently with ultrasound image acquisition. Blood pressure was recorded at least twice, and the average of these readings was calculated, following the International Consensus on Standardized Clinic Blood Pressure Measurement (13). All image acquisition and measurements strictly adhered to the guidelines established by the American Society of Echocardiography (14). In summary, after connecting the electrocardiogram leads, subjects were positioned in the left lateral decubitus position, and sequential views of the longitudinal, transverse, apical four-chamber, three-chamber, and two-chamber perspectives were obtained. Continuous data acquisition over five cardiac cycles at a frame rate of at least 60 frames per s resulted in standard two-dimensional grayscale dynamic images, which were stored in an offline workstation (EchoPAC version 204, GE Healthcare).

Measurements were taken in the parasternal long-axis view to assess the LV end-diastolic diameter (LVEDD), interventricular septal thickness in diastole (IVSD), and LV posterior wall thickness in diastole (LVPWD). The relative wall thickness (RWT) was calculated using the formula: 2 × LVPWD/LVEDD (15). The LV mass (LVM) was computed using the formula: LVM (g) = 0.8 × 1.04 × [(IVSD + LVPWD + LVEDD)^3^ − LVEDD^3^] + 0.6 (15). The LV mass index (LVMI) was calculated as the ratio of LVM to body surface area (BSA). According to the ASE/EACVI guidelines (14), the LV geometry of runners was classified into four categories: normal geometry (RWT ≤ 0.42 and without LVH), concentric remodeling (RWT > 0.42 and without LVH), concentric hypertrophy (RWT > 0.42 and with LVH), and eccentric hypertrophy (RWT ≤ 0.42 and with LVH).

The Simpson's biplane method was employed to measure LV and left atrial (LA) volumes, and the EchoPAC software automatically calculated the LV ejection fraction, stroke volume, and cardiac output. The LV sphericity index was calculated using the formula: LVEDV/(3/4 × *π* × [*D*/2]^3^), where LVEDV represents the LV end-diastolic volume, and D denotes the long-axis diameter at end-diastole as viewed in the apical four-chamber and two-chamber views (16). Doppler ultrasound was employed to measure the mitral peak *E* and *A*, along with the septal *e*′ velocity and lateral *e*′ velocity.

### 2.3 LV pressure-strain loop

**Figure 1:**
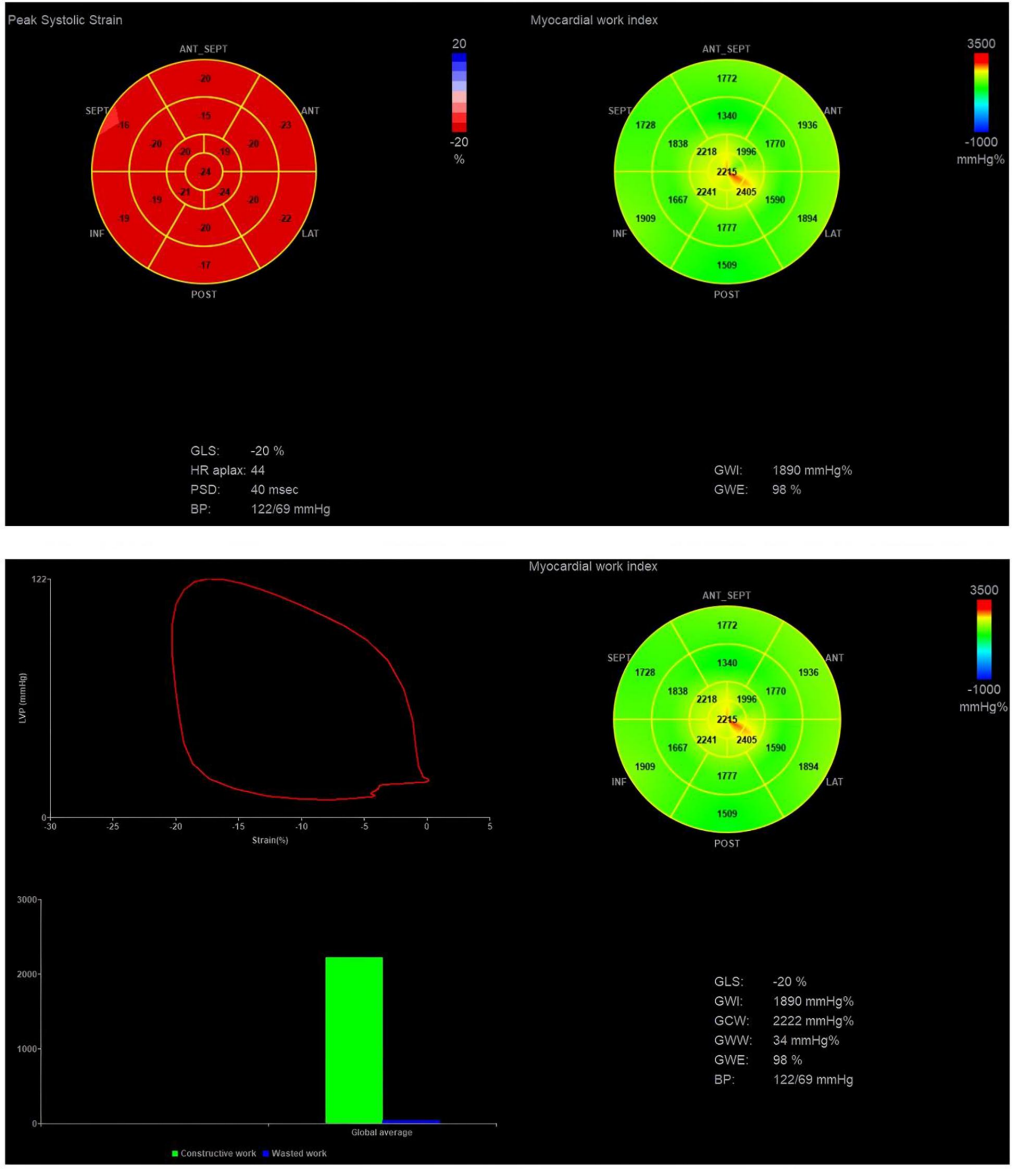
Pressure-strain loop analysis. GWI, global work index; GWE, global work efficiency; GCW, global constructive work; GWW, global wasted work.

The AFI module of the EchoPAC is utilized to analyze the pressure-strain loop (17). Dynamic images of the apical four-chamber, two-chamber, and three-chamber views are selected, with the software automatically identifying the boundaries of the LV endocardium and epicardium. It is crucial to ensure that all myocardium is included within the region of interest and to make manual adjustments as necessary. The software automatically calculates GLS and longitudinal peak strain dispersion throughout the entire cardiac cycle, then computes the average value from the three apical views. Non-invasive blood pressure measured during image acquisition is entered into the system, and the mitral and aortic valve closure and opening times are determined from the apical three-chamber view. As illustrated in Figure 1, the system automatically generates the pressure-strain loop. It computes the global work index (GWI), global constructive work (GCW), global wasted work (GWW), and global work efficiency (GWE).

GWI is the area of the pressure-strain loop, reflecting the total myocardial work from the closure to the opening of the mitral valve (11). GCW denotes the myocardial work that facilitates LV ejection, encompassing the positive shortening work during systole and the lengthening work during isovolumetric relaxation. GWW represents the myocardial work that impedes LV ejection, including the work of myocardial lengthening during systole and the work of myocardial shortening during isovolumetric relaxation. GWE is calculated using the formula GWE = GCW/(GCW + GWW) × 100%, which reflects the efficiency of myocardial work throughout the entire cardiac cycle (10).

### 2.4 Statistical analysis

Statistical analyses were conducted using SPSS version 26.0, while graphical representations were generated with GraphPad Prism version 8.0. The Shapiro–Wilk test was utilized to evaluate the normality of the data. Continuous variables were reported as mean and standard deviation [mean (SD)] or median and interquartile range [median (Q1–Q3)], depending on their distribution. Categorical variables were expressed as frequency and percentage. Comparisons between two groups were performed using either the independent samples *t*-test or the Mann–Whitney *U* test, contingent upon the normality of the data distribution. Intraclass correlation coefficients and coefficient of variation were used to assess the reproducibility of myocardial work parameters.

Given the relatively small sample size, a Firth logistic regression model was employed to examine the factors associated with LVH (18). This model is commonly used in studies with limited sample sizes because it produces bias-reduced effect estimates (19). The Hosmer–Lemeshow test was used to assess the model's goodness-of-fit. Additionally, Spearman correlation analysis was conducted to explore the relationship between running training and myocardial work. According to previous studies, correlation coefficient values less than |0.3| indicate a “small” effect size, values between |0.3| and |0.5| indicate a “moderate” effect size, and values greater than |0.5| indicate a “large” effect size (18). A *post hoc* analysis of the correlation was performed using PASS software (v15.0) with a two-tailed alpha of 0.05. It is noteworthy that *post hoc* power analysis estimates the test's power based on the observed effect size and sample size. The underlying idea is that non-significant results may arise from insufficient power (18).

A *p*-value of less than 0.05 (two-tailed) was considered statistically significant, and a 95% confidence interval (CI) was calculated. The R software (version 4.4.2), along with the logistf and ResourceSelection packages, was used to fit Firth penalized logistic regression and to perform the Hosmer–Lemeshow test.

## 3 Results

### 3.1 Demographic characteristics

Among the 68 marathon runners, 12 females and 14 males exhibited LVH. Demographic data for all participants are presented in Table 1. Compared to the control group, all runners exhibited significantly lower heart rates (*p* < 0.05). There was no significant difference in age between the control group and the runners. However, male runners had a significantly lower BMI than the control group (*p* < 0.05). Furthermore, female runners with LVH trained longer weekly distances, had more extensive long-distance running experience, and participated in a greater number of marathon races than the non-LVH group (*p* < 0.05). Male runners with LVH also trained longer weekly distances than the non-LVH group (*p* < 0.05).

**Table 1:** Characteristics of the study population stratified by sex.

Variable	Female-control	Female-runner (no LVH)	Female-runner (LVH)	Male-control	Male-runner (no LVH)	Male-runner (LVH)
N	19	14	12	21	28	14
Age (years)	42.4 (8.1)	41 (10.0)	43.0 (6.7)	40.7 (13.4)	40.5 (13.6)	39.1 (10.4)
Weight (kg)	55.6 (6.6)	55.6 (4.5)	55.7 (3.8)	71.9 (7.7)	63.4 (6.5)	67.1 (8.0)
Height (cm)	159 (4.2)	162 (4.0)	161 (4.7)	171 (5.9)	171 (6.6)	174 (6.6)
BMI (kg/m^2^)	22.1 (2.4)	21.1 (1.5)	21.4 (1.5)	24.5 (2.0)	21.5 (2.2)*	22.0 (1.6)*
BSA (m^2^)	1.56 (0.10)	1.58 (0.08)	1.58 (0.07)	1.84 (0.12)	1.74 (0.10)*	1.80 (0.14)
Heart rate (beats/min)	71.6 (10.4)	55.2 (9.0)*	56.4 (7.9)*	71.6 (8.5)	59.2 (8.6)*	57.5 (7.0)*
SBP (mmHg)	108 (11)	119 (13)*	115 (15)	120 (10)	124 (12)	125 (15)
DBP (mmHg)	76.9 (10.2)	83.4 (7.5)	78.8 (6.4)	81.7 (9.3)	82.0 (8.1)	82.1 (10.9)
Weekly average training (km)		50.3 (13.4)	66.4 (14.9)^†^		49.9 (12.6)	78.8 (13.1)^†^
Long-distance running experience (years)		3.0 (2.0–5.0)	7.5 (5.0–8.0)^†^		4.5 (2.0–7.0)	5.0 (3.0–8.3)
Number of previous marathons		9.0 (2.8–15.8)	24.0 (12.8–37.3)^†^		13.5 (4.0–29.5)	20.5 (13.3–33.8)

* *P* < 0.05 vs. Control.

† *P* < 0.05 vs. no LVH.

### 3.2 Echocardiographic measurements

The transthoracic echocardiographic parameters are presented in Table 2. Compared with the control group, runners exhibited significant increases in LV and LA volume index, interventricular septal and LV posterior wall thickness, LV mass index, and stroke volume (*p* < 0.05). However, these changes were more pronounced in runners with LVH, indicating a more severe degree of LV remodeling. No significant differences in LV ejection fraction were observed in runners. Regarding diastolic function, the male runners demonstrated a higher lateral mitral annular *e*′ velocity than the male controls.

**Table 2:** Echocardiographic measurements stratified by sex.

Variable	Female-control	Female-runner (no LVH)	Female-runner (LVH)	Male-control	Male-runner (no LVH)	Male-runner (LVH)
LA volume (mL)	36.7 (7.8)	46.6 (9.5)*	55.8 (7.3)^*^^,^^†^	41.3 (9.8)	49.1 (11.4)*	63.4 (11.5)^*^^,^^†^
LA volume index (mL/m^2^)	23.4 (4.5)	29.4 (5.4)*	34.0 (3.8)^*^^,^^†^	22.5 (5.9)	28.2 (6.1)*	35.1 (5.8)^*^^,^^†^
IVSD (mm)	8.8 (0.8)	9.3 (0.6)*	9.7 (0.5)*	9.5 (0.8)	9.9 (0.7)	11.0 (0.8)^*^^,^^†^
LVPWD (mm)	8.7 (0.7)	9.3 (0.6)*	9.5 (0.5)*	9.4 (0.8)	9.8 (0.7)*	10.6 (0.6)^*^^,^^†^
RWT	0.40 (0.03)	0.42 (0.05)	0.40 (0.03)	0.41 (0.03)	0.42 (0.04)	0.41 (0.04)
Geometry category (*N*, %)						
Normal	—	8 (57%)	—	—	13 (46%)	—
Concentric remodeling	—	6 (43%)	—	—	15 (54%)	—
Concentric LVH	—	—	4 (33%)	—	—	5 (36%)
Eccentric LVH	—	—	8 (67%)	—	—	9 (64%)
LVEDV (mL)	68.4 (11.2)	81.6 (10.5)*	87.6 (11.6)*	78.3 (16.0)	96.9 (22.3)*	124.2 (24.1)^*^^,^^†^
LVEDV index (mL/m^2^)	43.6 (51.5)	51.5 (5.8)*	55.4 (6.4)*	42.2 (7.7)	55.9 (11.9)*	63.1 (21.4)^*^^,^^†^
LV mass (g)	117 (16.9)	134 (13.3)*	163 (7.2)^*^^,^^†^	148 (28.0)	160 (22.3)*	218 (21.9)^*^^,^^†^
LV mass index (g/m^2^)	75.4 (10.0)	84.8 (7.1)*	103 (6.6)^*^^,^^†^	80.0 (12.6)	92.0 (10.4)*	120.9 (5.6)^*^^,^^†^
LVEF (%)	61.6 (4.4)	59.1 (3.6)	60.2 (5.9)	60.3 (4.6)	60.0 (3.7)	58.3 (3.8)
Cardiac output (L/min)	3.0 (0.7)	2.8 (0.6)	3.0 (0.6)	3.4 (0.6)	3.5 (0.9)	4.1 (1.1)^*^^,^^†^
Stroke volume (mL)	42.1 (6.8)	48.3 (6.9)*	52.7 (8.3)*	47.0 (8.6)	60.5 (12.7)*	72.1 (14.5)^*^^,^^†^
Spherical index	0.31 (0.05)	0.38 (0.07)*	0.38 (0.06)*	0.30 (0.05)	0.32 (0.06)	0.38 (0.06)^*^^,^^†^
Peak *E* (cm/s)	84.1 (12.7)	86.9 (16.5)	84.3 (16.3)	72.8 (12.6)	80.0 (17.5)	87.8 (15.4)*
Peak *A* (cm/s)	69.5 (16.2)	61.6 (7.8)	62.5 (9.6)	63.1 (14.2)	60.6 (12.2)	64.3 (9.9)
*E*/*A* ratio	1.26 (0.34)	1.42 (0.30)	1.36 (0.25)	1.21 (0.36)	1.38 (0.41)	1.37 (0.18)
Septal *e*′ (cm/s)	10.9 (2.4)	10.3 (2.5)	10.1 (1.5)	10.1 (2.4)	10.6 (2.6)	10.9 (1.9)
Lateral *e*′ (cm/s)	14.4 (2.1)	15.3 (2.8)	13.8 (3.7)	12.8 (2.8)	15.8 (3.8)*	15.0 (2.8)*
Average *E*/*e*′	6.8 (1.1)	6.9 (1.4)	7.2 (1.6)	6.5 (1.1)	6.2 (1.2)	6.9 (1.1)

IVSD, interventricular septum thickness at end-diastole; LVPWD, LV posterior wall thickness at end-diastole; LVEDV, LV end-diastolic volume; RWT, relative wall thickness. LA volume index and LVEDV index are calculated based on BSA.

* *P* < 0.05 vs. Control.

† *P* < 0.05 vs. no LVH.

Using LV and LA parameter thresholds categorized by sex, as outlined in the international guideline (14), we defined the LV and LA parameters in runners (Supplementary Figure 1). The results indicate that only a subset of amateur runners in this study exhibited abnormal LV and LA parameters. Interestingly, both male and female runners displayed a notable proportion of concentric remodeling and concentric LVH, characterized by relatively high RWT. When we applied the thresholds reported by Sheng et al. (20) for the Chinese population (Supplementary Table 1), the proportions of concentric remodeling and concentric hypertrophy in both male and female runners significantly decreased. This finding may suggest racial differences in RWT thresholds.

### 3.3 Pressure-strain loop

The pressure-strain parameters are summarized in Table 3. Compared with the female and male control groups, both female (mean, 1,902 vs. 1,707 mmHg%; *P* *<* 0.01) and male runners (mean, 1,842 vs. 1,679 mmHg%; *P* *<* 0.01) without LVH exhibit higher GWI. Additionally, male runners without LVH demonstrate a greater GCW (mean, 2,153 vs. 2,017 mmHg%; *P* *<* 0.01). Conversely, female and male runners with LVH do not show significant changes in GWI and GCW compared with the female and male control groups. Furthermore, no notable differences in GWI and GCW are observed between the LVH group and the non-LVH group. Interestingly, the GWE and GWW for both female and male runners with LVH are significantly lower than those recorded in the control group (*p* < 0.05). However, no significant differences in GWE or GWW are observed in male and female runners without LVH compared with the control group. These indicators suggest significant differences in myocardial work characteristics between runners with LVH and those without. Both female and male runners without LVH exhibit the highest myocardial work capacity and efficiency, whereas those with LVH experience a notable reduction in myocardial work efficiency. There were no significant differences in the GLS among the various groups. However, an increasing trend in PSD was observed in the LVH group, particularly among male runners with LVH. Furthermore, we compared the myocardial work of male and female runners with that of the healthy Chinese population reported by Wu et al. (21) (Supplementary Table 2). The results indicated that runners with LVH still exhibit relatively lower GWE and higher GWW.

**Table 3:** Pressure-strain loops parameters stratified by sex.

Variable	Female-control	Female-runner (no LVH)	Female-runner (LVH)	Male-control	Male-runner (no LVH)	Male-runner (LVH)
GWI (mmHg%)	1,707 (201)	1,902 (198)*	1,766 (260)	1,679 (213)	1,842 (210)*	1,770 (273)
GCW (mmHg%)	2,052 (251)	2,184 (176)	2,138 (347)	2,017 (231)	2,153 (233)*	2,079 (290)
GWW (mmHg%)	66.0 (55.0–77.0)	74.0 (50.3–98.7)	113.5 (93.3–128.0)^*^^,^^†^	68.0 (45.5–83.0)	57.5 (42.5–90.3)	108.0 (65.3–117.5)^*^^,^^†^
GWE (%)	96.0 (96.0–97.0)	96.0 (94.7–97.0)	94.5 (93.2–95.0)^*^^,^^†^	96.0 (95.5–97.0)	97.0 (95.0–97.0)	94.5 (94.0–96.0)^*^^,^^†^
GLS (%)	20.2 (2.1)	19.8 (1.3)	19.1 (2.2)	18.6 (1.28)	18.9 (1.61)	17.9 (1.64)
PSD (ms)	42.6 (9.3)	44.9 (6.4)	49.3 (7.0)*	39.8 (9.9)	42.6 (7.5)	45.1 (10.5)

GWI, global work index; GWE, global work efficiency; GCW, global constructive work; GWW, global wasted work; GLS, global longitudinal strain; PSD, peak strain dispersion.

* *P* < 0.05 vs. Control.

† *P* < 0.05 vs. no LVH.

### 3.4 Influencing factors for LVH in amateur marathon runners

We incorporated the characteristics of marathon running that may influence LVH into the Firth's penalized logistic regression model, using age and BSA as covariates and stratifying by gender. As shown in Table 4, weekly running distance (per 5 km) may be a factor affecting LVH in male runners (OR = 1.97, CI: 1.37–3.48). The Hosmer-Lemeshow test indicated that the regression model fit was adequate (*χ*^2^ = 7.66, *p* = 0.47). However, in female runners, the association between weekly running distance and LVH was not statistically significant (*p* = 0.08).

**Table 4:** Firth's penalized logistic regression model stratified by sex.

Variable	Male-runner				Female-runner			
	OR (95%CI)	*P*	Hosmer-Lemeshow test	*P*	OR (95%CI)	*P*	Hosmer-Lemeshow test	*P*
			*χ* ^2^				*χ* ^2^	
			7.66	0.47			6.85	0.55
Weekly average training (per 5 km)	1.97 (1.37–3.48)	<0.001			1.39 (0.96–2.29)	0.08		
Long-distance running experience (years)	1.21 (0.56–2.57)	0.62			1.38 (0.66–3.48)	0.40		
Number of previous marathons	1.03 (0.87–1.25)	0.73			0.97 (0.86–1.08)	0.61		

Age and BSA as covariates.

### 3.5 The relationship between weekly running distance and myocardial work

We analyzed the correlation between weekly running distance and myocardial work among all runners and stratified by gender (Table 5). In the overall cohort, weekly running distance showed a negative correlation with GWE (*r* = −0.37, *p* = 0.002) and a positive correlation with GWW (*r* = 0.34, *p* = 0.004), yielding *post hoc* power values of 0.82 and 0.80, respectively. Among female runners, weekly exercise distance showed a negative correlation with GWE (*r* = −0.49, *p* = 0.01) and a positive correlation with GWW (*r* = 0.51, *p* = 0.007), with *post hoc* power values of 0.66 and 0.71, respectively. In male runners, weekly exercise distance showed a negative correlation with GWE (*r* = −0.32, *p* = 0.04), whereas the correlation with GWW was not significant (*p* = 0.05), with *post-hoc* power values of 0.46 and 0.47, respectively.

**Table 5:** The correlation between myocardial work and the average weekly training distance.

Variable	All runner		Male-runner		Female-runner	
	*r* (95% CI)	*P*	*r* (95% CI)	*P*	*r* (95% CI)	*P*
GWI (mmHg%)	−0.09 (−0.33 to 0.15)	0.44	−0.08 (−0.38 to 0.24)	0.61	−0.02 (−0.42 to 0.38)	0.91
GCW (mmHg%)	−0.09 (−0.32 to 0.16)	0.47	−0.12 (−0.42 to 0.20)	0.45	0.02 (−0.38 to 0.42)	0.90
GWW (mmHg%)	0.34 (0.11 to 0.54)	0.004	0.30 (−0.01 to 0.56)	0.05	0.51 (0.14 to 0.76)	0.007
GWE (%)	−0.37 (−0.56 to −0.13)	0.002	−0.32 (−0.57 to −0.01)	0.04	−0.49 (−0.75 to −0.12)	0.01

GWI, global work index; GWE, global work efficiency; GCW, global constructive work; GWW, global wasted work.

### 3.6 Reproducibility

Previous studies have confirmed the excellent reproducibility of myocardial work parameters (21, 22). In our research, two observers conducted repeated analyses on 15 participants. The intra-observer intraclass correlation coefficients for GWI, GWE, GCW, and GWW were 0.974, 0.866, 0.972, and 0.979, respectively (*p* < 0.05). The inter-observer intraclass correlation coefficients for GWI, GWE, GCW, and GWW between observers were 0.972, 0.860, 0.968, and 0.977, respectively (*p* < 0.05). Additionally, the intra-observer coefficients of variation for GWI, GWE, GCW, and GWW were 1.7%, 0.3%, 1.8%, and 4.5%, respectively. The inter-observer coefficients of variation for GWI, GWE, GCW, and GWW were 1.8%, 0.4%, 1.8%, and 4.6%, respectively.

## 4 Discussion

To our knowledge, this study is the first to investigate the association between LVH and myocardial work in amateur runners. Our findings indicate that the GWE of runners with LVH is significantly reduced, while the GWW is significantly increased. This result suggests that LVH is associated with decreased myocardial work efficiency and increased myocardial wasted work in marathon runners. Additionally, we identified that weekly running distance is associated with LVH. An increase in weekly long-distance running may correlate with a reduction in GWE and an increase in GWW. These findings enhance the public's understanding of the impact of amateur marathon running on myocardial work and inform the development of appropriate training programs to mitigate potential adverse cardiac responses.

### 4.1 LVH in amateur marathon runners

In this study, the LA volume index, LV mass index, LV volume index, and wall thickness of female and male amateur runners were found to be significantly larger than those of the control group, which is consistent with prior research (23). The intense contraction of skeletal muscles during marathon running increases venous return to the heart, while sustained afterload overload contributes to LV cavity enlargement (24). Additionally, the thickening of the LV wall serves as a compensatory mechanism to maintain cardiac output (24). Compared to the normal thresholds in the ASE/EACVI guidelines (Supplementary Figure 1), a subset of amateur runners exhibited LA and LV characteristic abnormalities. The training intensity of amateur runners is generally lower than that of professional athletes, categorizing them in an intermediate range between the general population and elite athletes (25). Consequently, the cardiac characteristics of amateur runners may lie between those of the general population and professional athletes. Moreover, significant variations in training intensity among amateur runners may exist, with those training at higher intensities potentially exhibiting more pronounced heart abnormalities (23).

Interestingly, amateur runners in our study exhibited relatively high RWT, leading some runners to exhibit concentric hypertrophy, which contradicts previous understandings. However, Wilhelm et al. (26) reported RWT values of 0.41 for females and 0.44 for males among amateur runners, which are similar to our findings. The “Morganroth Hypothesis” posits that endurance exercise typically induces eccentric LVH due to volume overload, a theory that has been widely accepted (27). Nonetheless, some studies have questioned this hypothesis; for instance, Ryffel et al. (27) discovered that a portion of non-elite runners exhibited concentric hypertrophy. Trachsel et al. (28) found that using different classification methods resulted in significant variation in the proportion of concentric hypertrophy among amateur runners.

We attempted to geometrically classify the LV of amateur runners using RWT thresholds from other studies conducted in the Chinese population (20), and our results showed a notable change in the proportions of concentric hypertrophy. Additionally, we redefined the characteristics of the LV and LA using these thresholds, revealing discrepancies with the guideline thresholds, particularly pronounced in females (Supplementary Figure 1). This suggests that when applying guideline thresholds to define cardiac structures, it may be necessary to consider racial differences, normative thresholds, and variations in classification methods (20).

### 4.2 Myocardial work in amateur marathon runners

In our study, we found no significant differences in LVEF and GLS; however, notable changes in myocardial work were observed among amateur runners. Myocardial work integrates strain curves and blood pressure, accounting for myocardial deformation and afterload (10). Consequently, it may more accurately reflect intrinsic cardiac contractility in the context of exercise-induced LV remodeling (29). Guo et al. (30) also found that myocardial work can more accurately assess subclinical cardiac function in amateur runners than GLS.

We noted a trend of increasing GWI and GCW among amateur runners, particularly pronounced in those without LVH. This increase may be essential for maintaining adequate contractile function in response to increased volume load (31). Tokodi et al. (29) confirmed in animal experiments that GWI and GCW are significantly correlated with LV contractility measured via invasive catheterization, and they found elevated levels of GWI and GCW in swimmers. Furthermore, myocardial work is associated with peak oxygen consumption, suggesting that the increased GWI and GCW may enhance performance for amateur runners during exercise (29, 32).

Unlike previous studies, we found that individual differences exist in the myocardial work of amateur runners. Previous studies have reported decreases in GWE and increases in GWW among amateur runners (30). However, our research demonstrates that alterations in GWE and GWW are evident only in runners with LVH, whereas no such alterations are observed in those without LVH. The impact of LVH on myocardial function is significant (33). Among amateur runners, individuals with LVH show more pronounced LV wall thickening; however, because increased wall stress from volume overload may affect wall thickness, it may be greater at the base (30). This discordance may contribute to increased GWW and decreased GWE, indicating greater energy waste and reduced myocardial work efficiency. Therefore, we observed an upward trend in PSD among amateur marathon runners with LVH, consistent with a previous study in strength athletes (34). Furthermore, while increased afterload can enhance the performance of LV contraction, excessive LV remodeling may hinder the myocardium of amateur runners from generating effective work, leading to a greater amount of ineffective work.

### 4.3 The relationship between exercise volume, LVH, and myocardial work

The LVH in athletes is influenced by various factors, including the type of exercise, ethnicity, gender, and exercise volume (35). Our findings suggest that weekly running distance is associated with LVH among male runners. Compared with time and intensity, the weekly running distance provides a more accurate reflection of training levels (36). Prior research has indicated a direct relationship between endurance exercise training volume and LV mass, which enhances the aerobic capacity of amateur marathon athletes (37). Although the multiple regression model indicates that the correlation between female runners' weekly running distance and LVH is not significant at the 0.05 level, this may be due to the small sample size. The gender differences in the relationship between weekly running distance and LVH necessitate clarification through a larger sample study.

Our study reveals that weekly running distance is moderately correlated with GWE and GWW. As the weekly running distance increases, the GWE decreases, and the GWW gradually increases. This phenomenon may parallel the effects of medication; moderate exercise training is beneficial for the cardiovascular system, whereas excessive exercise may be detrimental (3). Champigneulle et al. (38) observed that myocardial performance in climbers initially improves and subsequently declines during exercise, suggesting that excessive training can increase myocardial ineffective work, thereby reducing myocardial work efficiency. This observation partially explains the cardiac fatigue phenomenon observed after marathon running (39).

We conducted a stratified analysis by gender; however, a *post hoc* power analysis revealed that the reduction in sample size due to gender stratification significantly reduced the power of the correlation results. Currently, there is no consensus on whether GWE and GWW are influenced by gender. Extensive clinical studies by Morbach et al. (40) and Galli et al. (41) suggest that GWE and GWW are not affected by gender; however, Olsen et al. (10) present a different perspective. To avoid overinterpretation, this study will refrain from discussing gender differences in GWE and GWW among runners. Additionally, we compared the myocardial work of runners with that of the healthy Chinese population reported by Wu et al. (21). The results indicate that runners with LVH exhibit lower GWE and higher GWW (Supplementary Table 2).

### 4.4 Study limitations

This study presents several limitations. First, the cross-sectional nature of the study precludes verification of causal relationships and longitudinal dose–response; therefore, we should interpret the association between training distance and myocardial work with caution. Subsequent longitudinal studies could investigate the dose-response relationship between exercise and myocardial work, as well as gender differences in this relationship. Second, the sample size is limited; we have conducted only a preliminary exploration of individual differences in myocardial work among amateur runners. Future research involving larger sample sizes and multi-center designs is necessary for further validation. Third, the population included in this study consists of amateur marathon runners, which may limit the applicability of our results to professional athletes. However, given the increasing popularity of marathon running, our findings may help the public develop effective moderate exercise training plans.

In addition, the pressure-strain model may be influenced by the software vendors and the methods of blood pressure measurement. The EchoPAC software used in this study is currently the most widely adopted commercial software, and blood pressure measurements adhere to international guidelines. Future studies could consider comparing the effects of different software vendors and various blood pressure measurement methods on the pressure-strain model. We selected two-dimensional echocardiography, the most commonly employed method in clinical practice, to assess cardiac dimensions and index the results by BSA. Future research could explore the use of three-dimensional echocardiography and incorporate allometric scaling or body composition metrics.

## 5 Conclusions

Amateur marathon runners without LVH exhibit higher myocardial work performance, while those with LVH demonstrate reduced myocardial work efficiency. Weekly training distance is associated with LVH in male runners. Additionally, a correlation exists between weekly training distance and the GWE and GWW. Therefore, marathon enthusiasts should develop moderate and individualized training plans to mitigate potential adverse cardiac responses.

## Data Availability

The original contributions presented in the study are included in the article/Supplementary Material, further inquiries can be directed to the corresponding authors.
