# Supplementary material for: Left ventricular hypertrophy and myocardial work in amateur marathon runners
Source: Front Cardiovasc Med. 2025 Dec 4;12:1707542. doi: 10.3389/fcvm.2025.1707542 (PMC12711797; doi:10.3389/fcvm.2025.1707542)
Supplement: Supplementary file 1 [file Datasheet1.pdf]

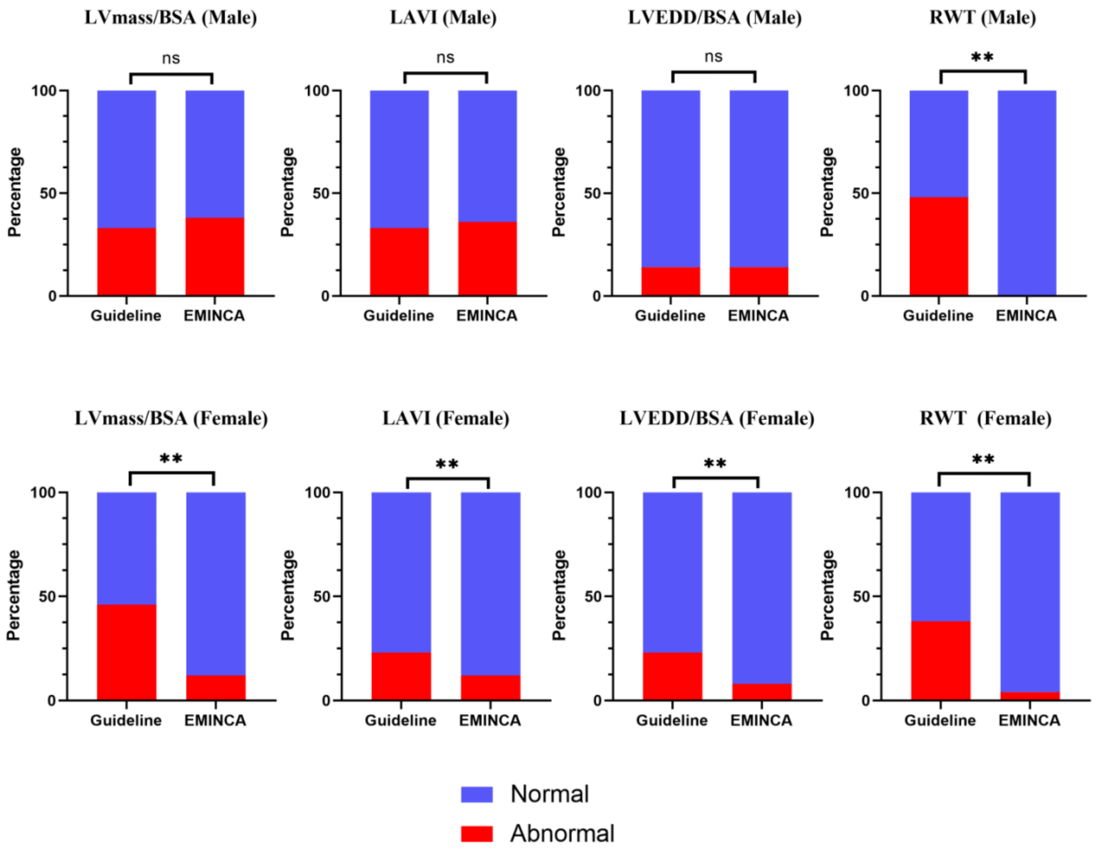

Supplementary Figure 1. Comparison of international guidelines and EMINCA data thresholds defining the left ventricle and left atrium in runners. BSA, body surface area; LAVI, left atrial volume/BSA; LV mass: left ventricular mass; RWT: relative wall thickness; LVEDD: left ventricular end-diastolic diameter; ns: no significance; **: $P < 0.01$. Guideline: ASE/EACVI international guidelines; EMINCA: an echocardiographic study involving 612 healthy Han Chinese adults.

Supplementary Table 1: Thresholds from ASE/EACVI international guidelines and EMINCA data.

	Female		Male	
	**Guidelines**	**EMINCA**	**Guidelines**	**EMINCA**
LV mass/BSA (g/m^2)	43–95	39–105	49–115	43–109
LAVI (mL/m^2)	9–33	10–37	16–34	16–34
LVEDD/BSA (mm/m^2)	23–31	22–32	22–30	20–30
RWT	0.25–0.51	0.24–0.42	0.25–0.51	0.24–0.42

BSA, body surface area; LAVI, left atrial volume/BSA; LV mass: left ventricular mass; RWT: relative wall thickness; LVEDD: left ventricular end-diastolic diameter. Guideline: ASE/EACVI international guidelines; EMINCA: an echocardiographic study involving 612 healthy Han Chinese adults.

Supplementary Table 2. Comparison of the runner's myocardial work in our study with that of the healthy population in other studies.

	Wu et al.	Our study		Wu et al.	Our study	
	Female healthy population	Female-Runner (no LVH)	Female-Runner (LVH)	Male healthy population	Male-Runner (no LVH)	Male-Runner (LVH)
Sample size	216	14	12	195	28	14
Country	China	China		China	China	
GWI (mmHg%)	1814 ± 228	1902±198	1766±260	1676 ± 211	1842±210	1770±273
GCW (mmHg%)	2085 ± 283	2184±176	2138±347	1946 ± 224	2153±233	2079±290
GWW (mmHg%)	74 (52–99)	74 (50-99)	114 (93-128)	81 (63–108)	58 (43-90)	108 (65-118)
GWE (%)	96 (95–97)	96 (95-97)	94 (93-95)	95 (94–96)	97 (95-97)	94 (94-96)

GWI: global work index; GCW: global constructive work; GWW: global wasted work; GWE: global work efficiency.
